# A risk analysis of alpelisib-induced hyperglycemia in patients with advanced solid tumors and breast cancer

**DOI:** 10.1186/s13058-024-01773-1

**Published:** 2024-03-04

**Authors:** Jordi Rodón, David Demanse, Hope S. Rugo, Howard A. Burris, Rafael Simó, Azeez Farooki, Melissa F. Wellons, Fabrice André, Huilin Hu, Dragica Vuina, Cornelia Quadt, Dejan Juric

**Affiliations:** 1 Division of Cancer Medicine, Department of Investigational Cancer Therapeutics, The University of Texas MD Anderson Cancer Center, 1515 Holcombe Blvd, Houston, TX 77030 USA; 2 Early Development Biostatistics, Novartis Pharma AG, Basel, Switzerland; 3 Division of Hematology and Oncology, Department of Medicine, University of California San Francisco Helen Diller Family Comprehensive Cancer Center, San Francisco, CA USA; 4 Department of Oncology, Sarah Cannon Research Institute, Tennessee Oncology Professional Limited Liability Corporation, Nashville, TN USA; 5 Diabetes and Metabolism Research Unit, Vall d’Hebron Research Institute, Barcelona, Spain; 6 Department of Medicine and Endocrinology, Autonomous University of Barcelona, Barcelona, Spain; 7 Centro de Investigación Biomédica en Red de Diabetes y Enfermedades Metabólicas Asociadas, Instituto de Salud Carlos III, Madrid, Spain; 8 Endocrinology Service, Department of Medicine, Memorial Sloan Kettering Cancer Center, New York, NY USA; 9 Vanderbilt University Medicine Center, Nashville, TN USA; 10 Department of Medical Oncology, INSERM U981, Gustave Roussy, Université Paris-Sud, Villejuif, France; 11 Novartis Pharmaceuticals Corporation, East Hanover, NJ USA; 12 Novartis Pharma AG, Basel, Switzerland; 13 Translational Clinical Oncology, Novartis Pharma AG, Basel, Switzerland; 14 Department of Medicine, Massachusetts General Hospital Cancer Center, Boston, MA USA

**Keywords:** Alpelisib, Hyperglycemia, Machine learning, SOLAR-1, BYLieve, HR+/HER2− advanced breast cancer

## Abstract

**Background:**

Hyperglycemia is an on-target effect of PI3Kα inhibitors. Early identification and intervention of treatment-induced hyperglycemia is important for improving management of patients receiving a PI3Kα inhibitor like alpelisib. Here, we characterize incidence of grade 3/4 alpelisib-related hyperglycemia, along with time to event, management, and outcomes using a machine learning model.

**Methods:**

Data for the risk model were pooled from patients receiving alpelisib ± fulvestrant in the open-label, phase 1 X2101 trial and the randomized, double-blind, phase 3 SOLAR-1 trial. The pooled population (*n* = 505) included patients with advanced solid tumors (X2101, *n* = 221) or HR+/HER2− advanced breast cancer (SOLAR-1, *n* = 284). External validation was performed using BYLieve trial patient data (*n* = 340). Hyperglycemia incidence and management were analyzed for SOLAR-1.

**Results:**

A random forest model identified 5 baseline characteristics most associated with risk of developing grade 3/4 hyperglycemia (fasting plasma glucose, body mass index, HbA_1c_, monocytes, age). This model was used to derive a score to classify patients as high or low risk for developing grade 3/4 hyperglycemia. Applying the model to patients treated with alpelisib and fulvestrant in SOLAR-1 showed higher incidence of hyperglycemia (all grade and grade 3/4), increased use of antihyperglycemic medications, and more discontinuations due to hyperglycemia (16.7% vs. 2.6% of discontinuations) in the high- versus low-risk group. Among patients in SOLAR-1 (alpelisib + fulvestrant arm) with *PIK3CA* mutations, median progression-free survival was similar between the high- and low-risk groups (11.0 vs. 10.9 months). For external validation, the model was applied to the BYLieve trial, for which successful classification into high- and low-risk groups with shorter time to grade 3/4 hyperglycemia in the high-risk group was observed.

**Conclusions:**

A risk model using 5 clinically relevant baseline characteristics was able to identify patients at higher or lower probability for developing alpelisib-induced hyperglycemia. Early identification of patients who may be at higher risk for hyperglycemia may improve management (including monitoring and early intervention) and potentially lead to improved outcomes.

*Registration:* ClinicalTrials.gov: NCT01219699 (registration date: October 13, 2010; retrospectively registered), ClinicalTrials.gov: NCT02437318 (registration date: May 7, 2015); ClinicalTrials.gov: NCT03056755 (registration date: February 17, 2017).

**Supplementary Information:**

The online version contains supplementary material available at 10.1186/s13058-024-01773-1.

## Background

A frequent mutation in hormone receptor–positive (HR+)/human epidermal growth factor receptor 2-negative (HER2−) breast cancer is alteration in the *PIK3CA* gene (encoding the phosphatidylinositol 3-kinase [PI3K] α isoform), which is mutated in approximately 40% of patients [1–4]. *PIK3CA* mutations are associated with poorer outcomes in metastatic breast cancer, promotion of tumor growth, and resistance to endocrine therapy (ET) [3, 5–7]. Targeted PI3K inhibition for patients with *PIK3CA* mutations has been explored as a pathway to overcome ET resistance in HR+breast cancers. Alpelisib, an orally available PI3K inhibitor that selectively inhibits and degrades the *α* isoform of PI3K, ultimately emerged as an agent that demonstrated compelling efficacy [8, 9].

Alpelisib has been approved by the US FDA and European Commission in combination with fulvestrant for the treatment of HR+/HER2− *PIK3CA*-mutated advanced or metastatic breast cancer following progression on or after ET [10, 11]. In the phase 3 SOLAR-1 trial of alpelisib in patients with HR+/HER2− advanced breast cancer (ABC) and prior ET, progression-free survival with alpelisib plus fulvestrant was significantly prolonged compared with fulvestrant alone (median, 11.0 vs. 5.7 months; HR, 0.65; 95% CI, 0.50–0.85; *P* < 0.001 [*PIK3CA*-mutated]) [12]. The most commonly reported grade 3/4 adverse event (AE) in the overall population was hyperglycemia (preferred term), which occurred in 36.6% of the alpelisib plus fulvestrant group vs 0.7% in the placebo plus fulvestrant group [12]. Due to the high incidence of grade 3/4 hyperglycemia, the SOLAR-1 protocol was amended to improve monitoring and management of hyperglycemia after enrollment began, resulting in improvements in markers of safety [13]. Hyperglycemia has also been previously reported in trials with other PI3K inhibitors, buparlisib and taselisib, in combination with fulvestrant [14, 15].

Due to the on-target nature of hyperglycemia for PI3K inhibitors, it is essential to understand the patient population at an elevated risk for hyperglycemia with alpelisib in order to provide timely interventions. This post hoc analysis data used a pooled population treated with alpelisib ± fulvestrant from the X2101 and SOLAR-1 trials and data collected during those respective trials to build a predictive model through machine learning techniques to identify patients who are more likely to develop grade 3/4 hyperglycemia, including early during treatment. This predictive model was then used to analyze risk in SOLAR-1. For an external validation, the model was applied to the BYLieve trial.

## Methods

### Study design and setting

Detailed study designs for X2101 (NCT01219699), SOLAR-1 (NCT02437318), and BYLieve (NCT03056755) have been described [12, 13, 16–18]. X2101 was a multicenter, open-label, phase 1 trial of alpelisib 30–450 mg (once daily)/120–200 mg (twice daily) as single agent or alpelisib 300–400 mg (once daily) plus fulvestrant [16, 17]. SOLAR-1 was a randomized, double-blind, placebo-controlled, phase 3 trial of alpelisib (300 mg) plus fulvestrant vs placebo plus fulvestrant [12]. BYLieve was an open-label, noncomparative, 3-cohort trial of alpelisib (300 mg) plus fulvestrant or letrozole [18]. All trials were approved by an independent ethics committee and institutional review board at each site and were conducted per the Declaration of Helsinki and Good Clinical Practice [12, 17]. All participants provided written informed consent, and participants in X2101 received financial compensation [12, 17, 18].

### Participants

X2101 enrolled patients with advanced solid tumors, including breast cancer, who had disease progression on, or could not tolerate, standard anticancer therapy or for whom no standard therapy existed. The majority of patients had a confirmed *PIK3CA* alteration. Patients with diabetes (fasting plasma glucose [FPG] ≥ 140 mg/dL [7.8 mM]) or a history of gestational or steroid-induced diabetes were excluded [16, 17].

Patients in SOLAR-1 had locally confirmed HR+/HER2− ABC and were eligible for further ET following relapse or progression. Patients were receiving or had previously received an aromatase inhibitor (AI) as neoadjuvant or adjuvant therapy or for advanced disease. Patients with type 1 or type 2 diabetes (FPG > 140 mg/dL [7.8 mM] or glycated hemoglobin [HbA_1c_] > 6.4%) were excluded.

Patients in BYLieve had HR+/HER2− ABC and confirmed *PIK3CA* mutation in tumor tissue or plasma. Patients were required to have CDK4/6 inhibitor plus AI, CDK4/6 inhibitor plus fulvestrant, or either chemotherapy or ET as last therapy and to have an FPG of ≤ 140 mg/dL (7.8 mM) and HbA_1c_ of ≤ 6.4% [18].

### Procedures

#### Statistical modeling of grade 3/4 hyperglycemic events

The hyperglycemia risk model was built using patients pooled from X2101 (alpelisib ± fulvestrant) and SOLAR-1 (alpelisib and fulvestrant arm only) in a post hoc analysis of hyperglycemic events. The pooled population was subdivided into a training set (405 patients [80%]) for model development and selection, a testing set (100 patients [20%]), and a validation set for model performance and validation (Additional file 1: Figure S1). The main objective of the statistical modeling was to minimize the null deviance residuals using a simple intercept Cox model using the time to first grade 3/4 hyperglycemia in patients treated with alpelisib ± fulvestrant [19]. Additional details are provided in Additional file 1: Supplemental Methods.

#### Analysis of high- and low-risk patients with hyperglycemia in SOLAR-1 and BYLieve

The final machine learning model was applied to the alpelisib plus fulvestrant arm of the SOLAR-1 trial to classify patients into low- and high-risk groups. Further analysis on clinically relevant outcomes was performed on SOLAR-1, which included only patients with ABC (the approved indication for alpelisib). For external validation, the final model was used to analyze grade 3/4 hyperglycemia in BYLieve (all cohorts). Time to onset of CTCAE grade 3/4 AEs was defined as the time from the start of treatment to the first incidence of a grade 3/4 hyperglycemia event. In the absence of an event during the on-treatment period, the censoring date applied was the earliest of the following dates: end date of on-treatment period (end of study treatment plus 30 days), death date, start date of new antineoplastic therapy (with the exception of palliative radiotherapy or fulvestrant monotherapy) before experiencing a CTCAE grade ≥ 3 event, data cutoff date, or date of withdrawal of informed consent.

Among patients from SOLAR-1 treated with alpelisib and fulvestrant, those with *PIK3CA* mutations were included in efficacy (progression-free survival [PFS]) analysis of high- and low-risk patients. PFS was defined as time from the date of randomization to the date of the first documented progression or death due to any cause. PFS times were censored if no PFS event was observed before the data cutoff.

## Results

### Patient population and hyperglycemia events

**Fig. 1:**
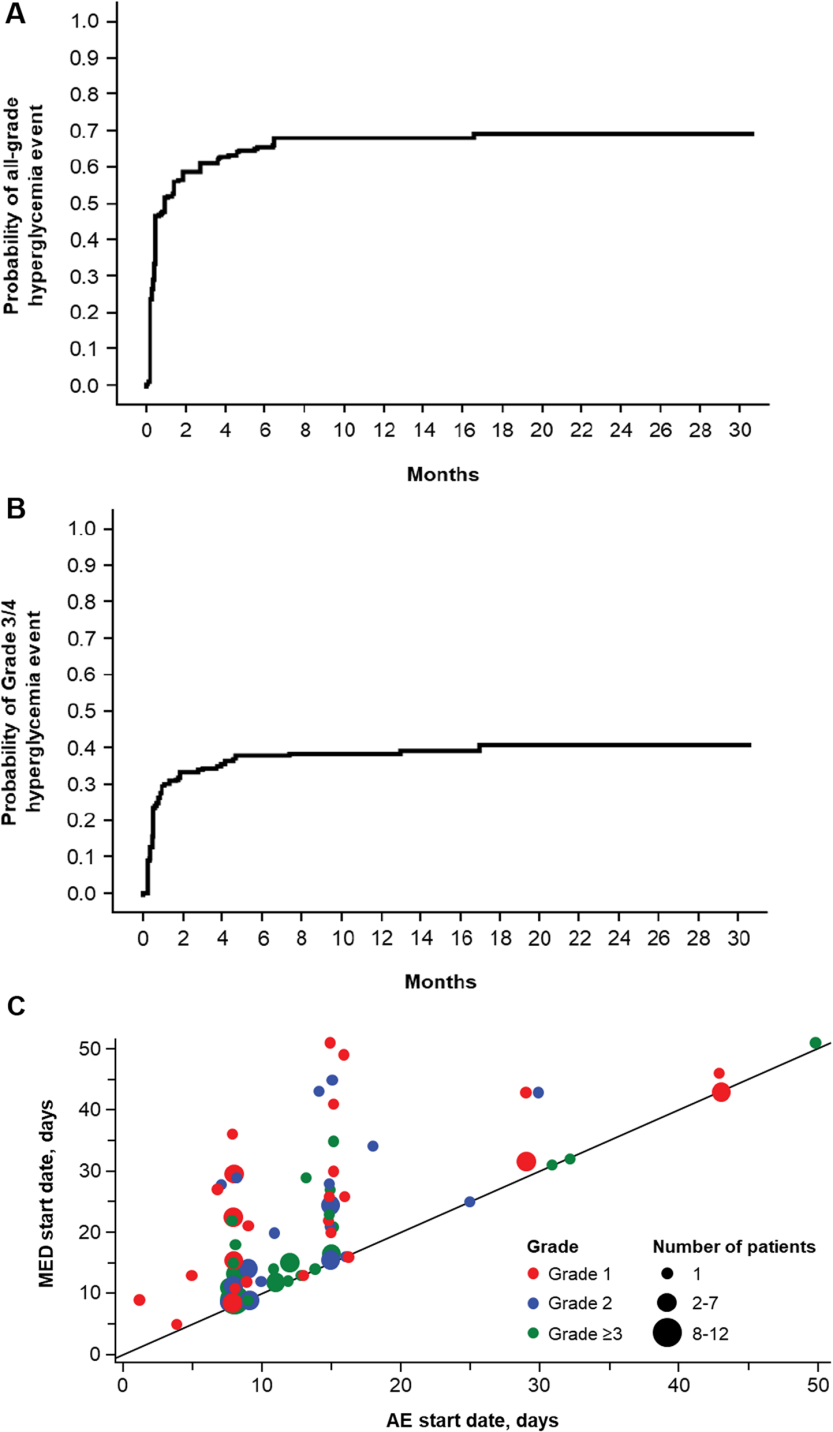

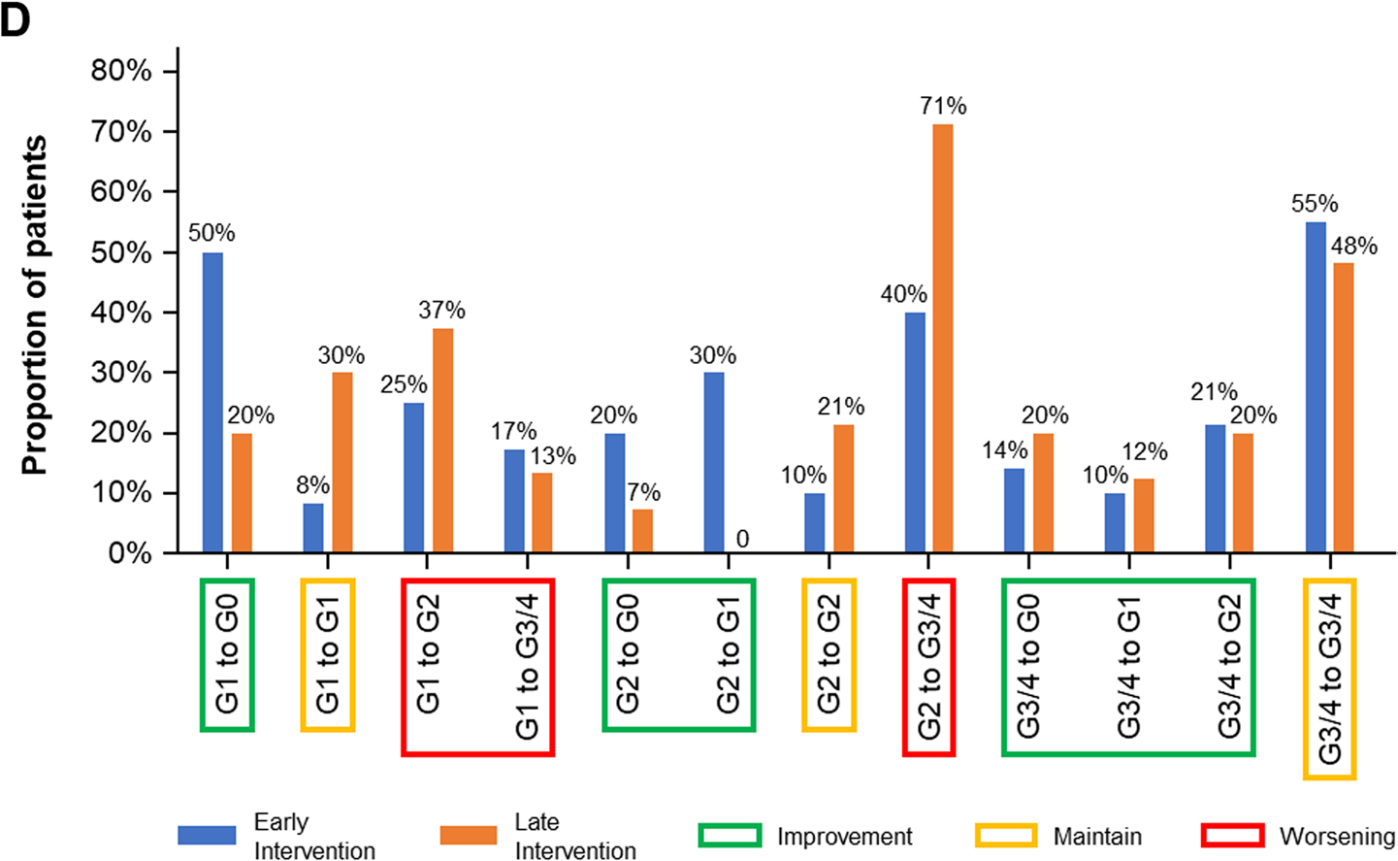
Cumulative incidence of all-grade hyperglycemic events among patients in SOLAR-1 treated with alpelisib + fulvestrant (**A**) or grade 3/4 hyperglycemic events among patients in SOLAR-1 treated with alpelisib + fulvestrant (**B**). Time to first medication vs first hyperglycemic event in patients treated with alpelisib from SOLAR-1 who had a hyperglycemia adverse event and was treated by antihyperglycemic medication (**C**) and subsequent grade of hyperglycemia among patients in SOLAR-1 with early (first quantile of time from hyperglycemia to treatment) and late (fourth quantile) treatment with antihyperglycemic medication (**D**). Cumulative incidence curves using Kaplan–Meier method. Hyperglycemic events by Standardized MedDRA query. *AE*, adverse event; *G*, grade

Baseline characteristics from both the X2101 (221 patients, data as of March 22, 2017) and SOLAR-1 trials (284 alpelisib plus fulvestrant patients, data as of June 12, 2018) were previously reported [12, 16, 17]. The median time to onset of grade ≥ 3 hyperglycemia (SOLAR-1 patients treated with alpelisib) was 15 days. Among the patients in the analysis from the SOLAR-1 trial, all-grade and grade 3/4 hyperglycemia (grouped terms) occurred in 187/284 (65.8%) and 108/284 (38.0%) patients, respectively (Fig. 1A and B). Of the patients from SOLAR-1 who developed hyperglycemia, 163/187 (87.2%) required a concomitant medication. One antihyperglycemic medication was required in 67/187 (35.8%) patients, while 49/187 (26.2%) and 47/187 (25.1%) required 2 and ≥ 3, respectively; metformin was the most common medication (142/163 [87.1%]), followed by insulin (52/163 [31.9%]). Plotting time to first event against time to first antihyperglycemic medication indicates that there were delays in treatment of hyperglycemia in some patients, even when hyperglycemia was identified early (Fig. 1C). A greater proportion of patients who received early intervention (first quantile for time from hyperglycemia to antihyperglycemic medication) for grade 1/2 hyperglycemia had improvement to a lower grade vs patients who received later intervention (fourth quantile) (Fig. 1D). Discontinuations due to hyperglycemia occurred in 19/284 patients (6.7%). Among patients in the BYLieve trial, metformin was given to 56/58 (96.6%) of patients who received an antihyperglycemic medication in Cohort A, 65/68 (95.6%) in Cohort B, and 61/66 (92.4%) in Cohort C. Insulin was given to 21/58 (36.2%) in Cohort A, 17/68 (25.0%) in Cohort B, and 18/66 (27.3%) in Cohort C.

### Model development

Model development using the training set was conducted to better identify patients at high risk for grade 3/4 hyperglycemic events. From a set of 36 variables, univariate analysis identified 13 variables for model building, defined by an adjusted *P* < 0.1 (Table 1). According to β coefficient, the most influential, significant parameters for predicting grade 3/4 hyperglycemia in the univariate analysis were FPG and body mass index (BMI); these were also the most important parameters identified by the elastic net model and linear model with stepwise variable selection. Based on the variables identified in the univariate analysis, 6 models were investigated for classification of risk of a grade 3/4 hyperglycemia event (Additional file 1: Figure S2). The area under the curve (AUC) over time in the training and test sets was used to compare the accuracy of the model scores at distinguishing patients who will develop from those who are not likely to develop a grade 3/4 hyperglycemia event. Model 4, the random forest model, performed notably better than the other models.

**Table 1:** Variables and corresponding β coefficients of the univariate, elastic net, and stepwise regression models in the training set

Variable	β univariate model^a^	*P*	Adjusted *P*^b^	β elastic net model (model 1)	β linear model with stepwise variable selection (model 2)
Age	0.182	< 0.001	0.003	0.024	NA
bid dosing	0.394	0.132	0.132	0.041	0.084
BMI	0.307	< 0.001	< 0.001	0.164	0.198
C-peptide	0.116	0.036	0.047	0.000	NA
Diastolic blood pressure	0.162	0.003	0.007	0.078	0.125
Dose	0.088	0.110	0.119	0.037	0.082
HbA_1c_	0.202	< 0.001	0.001	0.066	0.104
HDL cholesterol	− 0.099	0.073	0.086	0.000	NA
Monocytes	0.125	0.024	0.035	0.047	0.073
Fasting plasma glucose	0.440	< 0.001	< 0.001	0.321	0.357
Red blood cells	0.128	0.021	0.034	0.012	NA
Systolic blood pressure	0.174	0.002	0.004	0.000	NA
Triglycerides	0.142	0.010	0.019	0.000	NA

^a^Positive coefficients are associated with higher risk; negative coefficients are associated with lower risk. All variables were standardized to have a mean of 0 and an SD of 1 using training set data. ^b^Adjusted *P* values for simple multiple testing procedures using Benjamini and Hochberg step-up false discovery rate-controlling procedure

*bid*, twice daily; *BMI*, body mass index; *HbA*_*1c*_, glycated hemoglobin; *HDL*, high-density lipoprotein; *NA*, not applicable

**Fig. 2:**
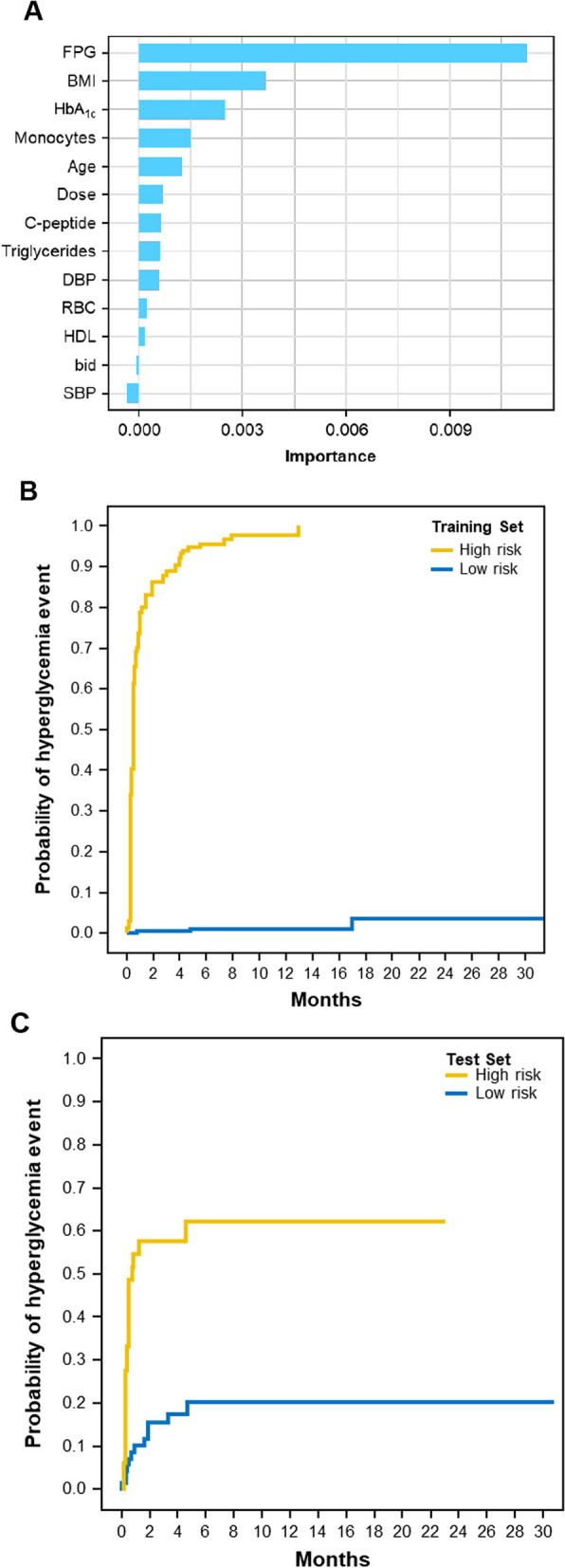
Variable importance of random forest (model 4) in the X2101 + SOLAR-1 training set (**A**) and prognostic association of risk status based on a random forest model with fasting plasma glucose, BMI, HbA_1c_, monocytes, and age (model 7) with time to grade 3/4 hyperglycemia event in the X2101 + SOLAR-1 training set (**B**) and the X2101 + SOLAR-1 test set (**C**). **A** was calculated by conditional permutation importance, **B** was calculated by cumulative incidence curves, and **C** was calculated using the Kaplan–Meier method. *bid*, twice daily dosing; *BMI*, body mass index; *DBP*, diastolic blood pressure; *FPG*, fasting plasma glucose; *HDL*, high-density lipoprotein cholesterol; *RBC*, red blood cells; *SBP*, systolic blood pressure.

For developing a clinically relevant simplified random forest model, a conditional permutation importance analysis was performed to identify the relative importance of the variables in model 4 (Fig. 2A). Model 7, the simplified random forest, was generated using only the 5 most influential variables from model 4: FPG, BMI, HbA_1c_, monocytes, and age. The AUC over time was similar for models 4 and 7 in the training and test sets (Additional file 1: Figure S2). At 2 months, the AUCs for models 4 and 7 were similar in the training set (0.994 and 0.991, respectively) and the test set (0.729 and 0.767, respectively). Patients were classified into high- or low-risk groups for grade 3/4 hyperglycemia based on the predictive risk score using model 7. In the training set, there was clear classification between the low- and high-risk groups for the probability of grade 3/4 hyperglycemia events (Fig. 2B). In the high-risk group, there was an 86.2% risk of a grade 3/4 event by 2 months’ treatment; there was a < 5% probability of an event in the low-risk patients over 30 months (Fig. 2B, Additional file 1: Table S1). In the test set, the high-risk group had approximately 57.6% chance of a grade 3/4 hyperglycemia event by month 2, while the low-risk patients had < 20% probability of developing grade 3/4 hyperglycemia over 30 months (Fig. 2C, Additional file 1: Table S1). The median (95% CI) time to grade 3/4 hyperglycemia events in the high-risk group was 15 (13–15) days in the training set and 20 (15-NE) days in the test set (Additional file 1: Table S1).

Using model 7, individual scores could be derived for a patient at baseline in order to determine the corresponding risk of a hyperglycemia event at a given time (Additional file 1: Figure S3). The density of individual scores for patients in the training set showed a bimodal distribution of high- and low-risk groups. In the pooled studies (n = 505), compared with low-risk patients, high-risk patients at baseline were more likely to be obese (BMI ≥ 30 kg/m^2^; 37.2% high risk vs. 15.0% low risk, respectively), have higher FPG (≥ 5.6 mmol/L; 71.3% vs. 22.6%), and have higher HbA_1c_ (≥ 5.7%; 56.7% vs. 27.6%) (Additional file 1: Table S2).

### Model application

Applying model 7 to the alpelisib arm of SOLAR-1 resulted in 106 patients in the high- and 178 in the low-risk groups. All-grade hyperglycemia occurred in 101/106 (95.3%) high- and 86/178 (48.3%) low-risk patients; grade 3/4 events occurred in 96/106 (90.6%) and 12/178 (6.7%), respectively. Antihyperglycemic medication was used in 94/106 (88.7%) high- and 70/178 (39.3%) low-risk patients. The majority of high-risk patients who received an antihyperglycemic medication required ≥ 3 (39/94 [41.5%]), followed by 2 (31/94 [33.0%]), and 1 medication (24/94 [25.5%]). Conversely, the majority of low-risk patients only required 1 antihyperglycemic medication (43/70 [61.4%]), followed by 2 (18/70 [25.7%]) and ≥ 3 medications (9/70 [12.9%]).

Alpelisib discontinuations were similar overall (90/106 [84.9%] vs. 154/178 [86.5%] for high vs low); however, discontinuation due to AE was more common in the high-risk (32/90 [35.6%]) vs. low-risk (39/154 [25.3%]) group, while discontinuation due to progressive disease was more common in the low-risk group (46/90 [51.1%] vs. 91/154 [59.1%] for high vs. low). Among patients who discontinued alpelisib, there were more discontinuations due to hyperglycemia in the high- vs. low-risk group (15/90 [16.7%]) vs. 4/154 [2.6%]). Dose reductions (85/106 [80.2%] vs. 83/178 [46.6%]) and interruptions (89/106 [84.0%] vs. 116/178 [65.2%]) were more common in the high- vs low-risk groups, respectively. Median relative dose intensity for high- vs. low-risk group was 75% vs. 93%, with median dose intensities of 223.9 vs. 278.8 mg/day, respectively.

**Fig. 3:**
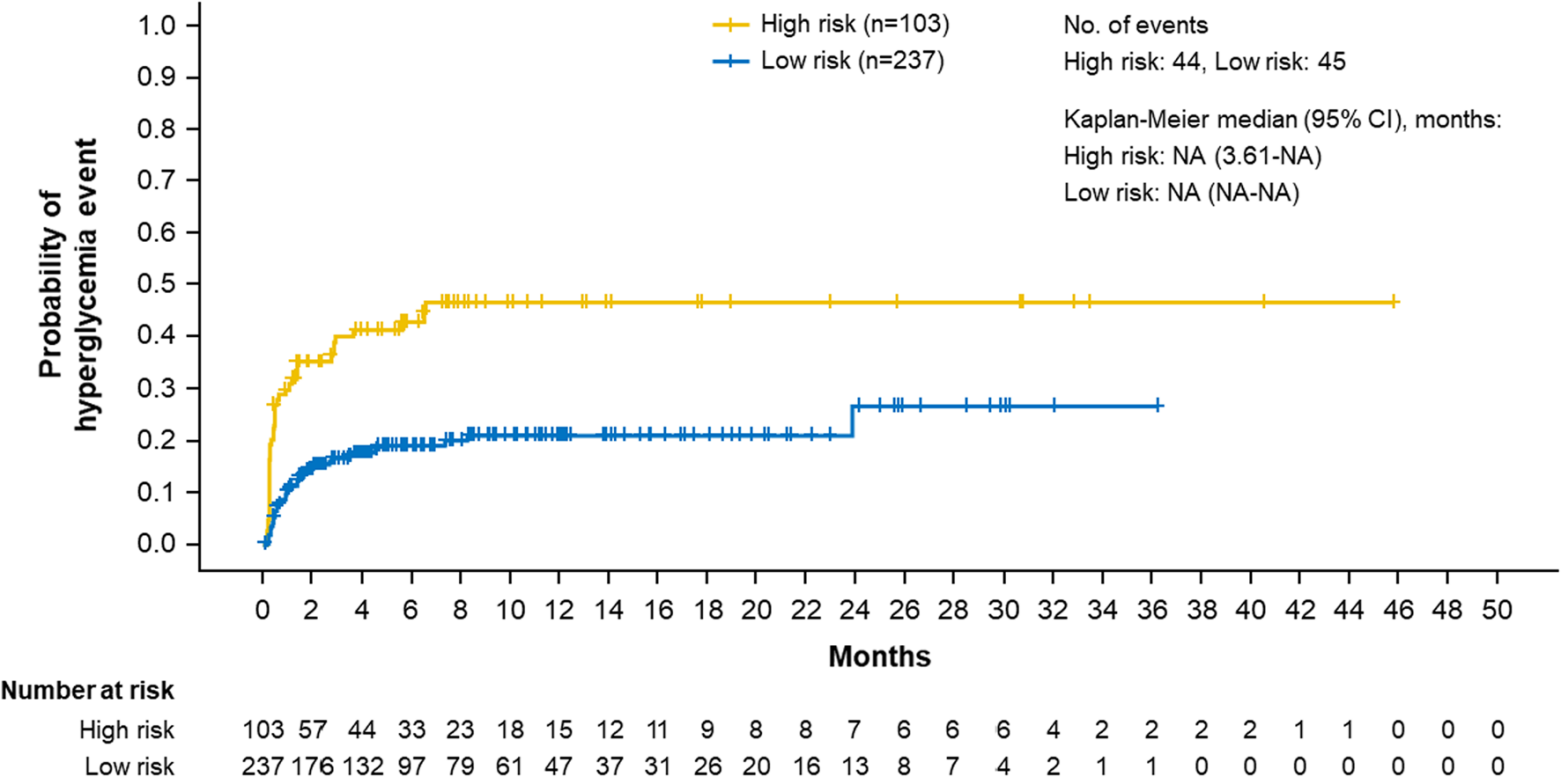
Time to grade 3/4 hyperglycemia in patients classified as high or low risk by model 7 in BYLieve

For external validation, model 7 was applied to all patients in BYLieve, resulting in 103 vs. 237 patients in the high- vs low-risk categories. Consistent with the results observed in SOLAR-1, a significant difference was observed in time to grade 3/4 hyperglycemia favoring low-risk patients (HR, 0.367; 95% CI, 0.241–0.558; *P* < 0.0001) (Fig. 3).

**Fig. 4:**
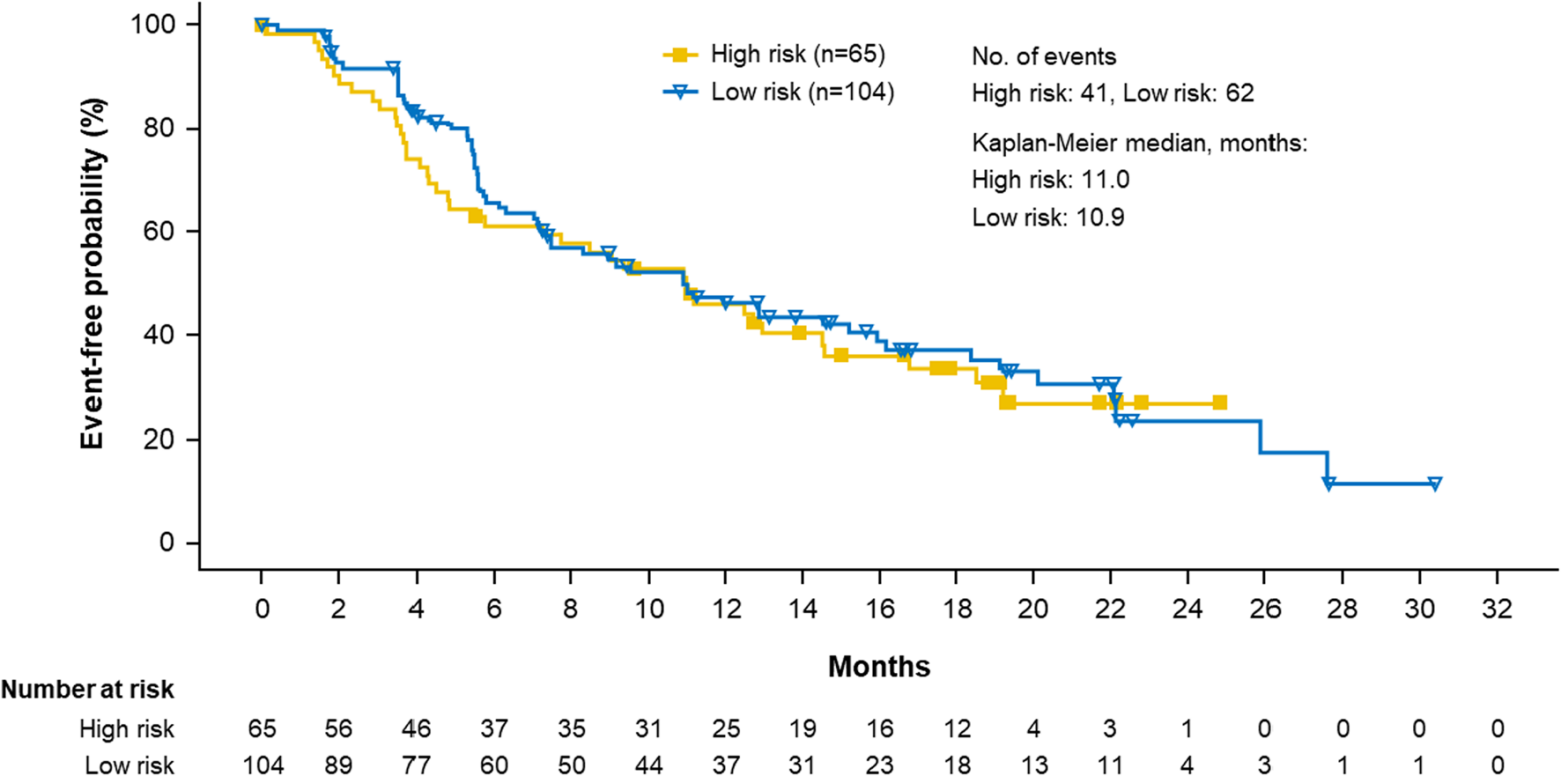
Progression-free survival for patients with *PIK3CA* mutations with high and low risk of hyperglycemia (model 7) treated with alpelisib in SOLAR-1

Efficacy analysis among patients in SOLAR-1 with a *PIK3CA* mutation who were treated with alpelisib + fulvestrant showed Kaplan–Meier PFS curves with a slight separation prior to 6 months, after which the curves appeared to converge for the remaining time points. The median PFS was similar between high- (11.0 months) and low-risk patients (10.9 months; HR, 1.097; 95% CI, 0.733–1.641) (Fig. 4).

## Discussion

As previously reported, hyperglycemia, an on-target effect of PI3Kα inhibition, was the most common all-grade and grade 3/4 AE in the alpelisib arm of SOLAR-1 [12, 13]. Of the patients with a hyperglycemia event, the majority had quick onset of event and required intervention with an antihyperglycemic medication. Development of a statistical model using data from the X2101 and SOLAR-1 trials resulted in an individual risk score that can be used to identify patients with high/low risk of experiencing early grade 3/4 hyperglycemia upon receiving alpelisib. The model used 5 clinically relevant variables that the majority of physicians are already collecting at baseline. When applying this model to the alpelisib arm of SOLAR-1 and to all patients in BYLieve, high-risk patients had a higher incidence of grade 3/4 hyperglycemia. High-risk patients in SOLAR-1 required more antihyperglycemic medications and had more hyperglycemia-related discontinuations than the low-risk patients.

Despite the differences in hyperglycemia incidence and management in SOLAR-1, similar median PFS was observed among high- and low-risk patients with *PIK3CA* mutations treated with alpelisib + fulvestrant (median PFS, 11.0 vs 10.9 months; HR, 1.097; 95% CI, 0.733–1.641). This may be unexpected given that the high-risk group had a higher percentage of dose reductions and interruptions, as well as lower dose intensity, than the low-risk group. Indeed, a prior analysis of PFS by median dose intensity in the SOLAR-1 trial showed a numerically lower median PFS for patients with a median dose intensity of < 248 versus ≥ 248 mg/day (9.6 vs. 12.5 months) [13]. The exact reasons for the lack of difference in PFS between the 2 groups in this analysis is unclear. However, there is a separation of the Kaplan–Meier curves prior to ≈ 6 months, during which the high-risk group appears to have worse PFS. This early separation could be related to the early-onset nature of the hyperglycemia; it would be during this period that most of the dose modifications would be expected to occur, along with the corresponding impact on PFS. This analysis is post hoc in nature, and the classification of the 2 groups may have resulted in imbalances in patient characteristics that could have impacted PFS. Therefore, the full effects of hyperglycemia on efficacy may be difficult to determine based on this PFS analysis alone. Additional analyses need to be done to further investigate the relationship between hyperglycemia, dose modification, and efficacy in these patients, including analyses to address potential immortal time bias.

Developing a model with clinical utility is important for the early identification of patients who may have a higher probability of a grade 3/4 hyperglycemia event while receiving alpelisib. Elevated FPG and HbA_1c_ at baseline are expected characteristics that would increase the probability of developing grade 3/4 hyperglycemia while receiving alpelisib. Similarly, higher BMI (> 30) and advanced age (> 75 years) have been previously reported as risk factors for alpelisib-induced hyperglycemia [13]. Monocytes, however, may be an unexpected influential baseline factor for predicting the likelihood of hyperglycemia. Monocytes may be elevated in patients who are obese (with or without diabetes), and previous studies have shown that proinflammatory monocytes might be associated with metabolic syndromes [20, 21]. Identification of monocytes as an important factor in determining risk of hyperglycemia warrants further investigation.

While applying this model to the alpelisib arm of SOLAR-1 helps to confirm the function of the risk model, it is important to understand the context of hyperglycemia management within the trial. Monitoring and treatment guidelines for hyperglycemia were being optimized during SOLAR-1. Initially, a check for FPG was required at day 15, which we now know is the median time to onset in the trial. This means that approximately half of patients who developed hyperglycemia did so prior to the first control. After enrolling approximately 50% of patients, a protocol amendment was introduced detailing recommendations for improved monitoring and management of hyperglycemia [13]. Rugo et al. [13] showed that implementation of these guidelines helped prevent alpelisib discontinuations and limited dose adjustments. However, many patients still had delayed intervention even after identification of hyperglycemia (Fig. 1C), sometimes leading to higher grades, dose adjustments, and discontinuations of alpelisib. Therefore, some of the incidence and management data in the high-risk patient group may be impacted in part by evolving treatment guidelines during SOLAR-1. Earlier identification of patients and timely intervention for alpelisib-induced hyperglycemia in both high- and low-risk patients may lead to further improvement of hyperglycemia management than indicated by the SOLAR-1 data. Indeed, the METALLICA trial tested prophylactic metformin administered with fulvestrant + alpelisib in patients with normal blood glucose at baseline or those who had prediabetes. METALLICA met its primary endpoint and showed that prophylactic metformin resulted in lower incidences of grade 3/4 hyperglycemia in both cohorts (normal blood glucose or prediabetes) compared with what was observed in corresponding patients in SOLAR-1 and BYLieve [22]. There were no discontinuations due to hyperglycemia in METALLICA.

## Conclusions

A risk model identifying 5 baseline factors (FPG, BMI, HbA_1c_, monocytes, and age) was able to classify patients receiving alpelisib in SOLAR-1 into high and low risk for grade 3/4 hyperglycemia. While treatment guidelines in SOLAR-1 were optimized during the course of the study, high-risk patients still demonstrated a higher incidence of all-grade and grade 3/4 hyperglycemia and increased use of antihyperglycemic medications compared with low-risk patients. These findings indicate that this clinically relevant risk model predicts the probability of alpelisib-induced grade 3/4 hyperglycemia using baseline factors. Earlier identification and intervention for treatment-induced hyperglycemia can potentially improve clinical management and lead to better outcomes for patients with HR+/HER2− ABC.

### Supplementary Information

**Additional file 1.** Supplemental Appendix.
