# Supplementary material for: A risk analysis of alpelisib-induced hyperglycemia in patients with advanced solid tumors and breast cancer
Source: Breast Cancer Res. 2024 Mar 4;26:36. doi: 10.1186/s13058-024-01773-1 (PMC10913434; doi:10.1186/s13058-024-01773-1)
Supplement: Supplementary file 1 — Additional file 1. Supplemental Appendix. [file 13058_2024_1773_MOESM1_ESM.docx]

SUPPLEMENTAL APPENDIX

**Supplemental Methods**

Procedures

In X2101, routine laboratory assessments were conducted at baseline, weekly until day 28 of cycle 2, and then at 2-week intervals. After 6 cycles of treatment were completed, day 15 visits were no longer required. Adverse events (AEs) were graded using Common Terminology Criteria for Adverse Events (CTCAE) v4.0. In SOLAR-1, routine laboratory assessments were conducted at screening, every 2 weeks for the first 8 weeks, and then every 4 weeks; fasting plasma glucose was assessed on day 8. In BYLieve, fasting or random blood glucose was analyzed on days 8 and 15 of cycle 1, days 1 and 15 of cycle 2, and day 1 of subsequent cycles. AEs in SOLAR-1 and BYLieve were recorded continuously using CTCAE v4.03 until 30 days after the final dose of trial treatment. A protocol amendment outlining AE management recommendations for hyperglycemia in SOLAR-1 was previously summarized.^15^ Alpelisib dose adjustments or interruptions were allowed for the management of hyperglycemia in all 3 trials. For these analyses, hyperglycemic events were defined by a standardized MedDRA query of preferred terms related to hyperglycemia.

An initial set of 48 and 42 baseline variables were identified in X2101 and SOLAR-1, respectively. Thirty-six baseline covariates that were included in both trials and had less than 20% missing data were investigated in a univariate regression analysis in the training set. The included variables were age, body mass index, albumin, alkaline phosphatase, alanine aminotransferase, aspartate aminotransferase, basophils, total bilirubin, calcium, total cholesterol, creatinine, eosinophils, fasting plasma glucose, glycated hemoglobin, hematocrit, high-density lipoprotein cholesterol, hemoglobin, potassium, lipase, lymphocytes, magnesium, monocytes, sodium, neutrophils, platelets, red blood cells, total protein, triglycerides, white blood cells, dose (alpelisib dose in mg), C-peptide, diastolic blood pressure, systolic blood pressure, neutrophil-to-lymphocyte ratio, platelets-to-lymphocyte ratio, and twice-daily dosing (bid regimen yes/no). All variables were standardized to have a mean of 0 and an SD of 1 using training set data. *P* values were adjusted for simple multiple testing procedures using Benjamini and Hochberg step-up false discovery rate (FDR) controlling procedure. The baseline covariates selected from the univariate regression analysis while controlling FDR at 0.1 or known to be part of the metabolic syndrome were used to develop supervised machine learning (ML) models, including stepwise regression, elastic net, random forest, gradient-boosted decision trees, and neural network. A 2-fold cross-validation was repeated 20 times to tune the hyperparameters. The final ML model was selected based on the performance in the training set using the time-dependent receiver operating characteristic curves and was also assessed in the test set.[23] A reduced ML model was considered more suitable for clinical practice. This model was used to derive a composite continuous risk score value. To facilitate clinical interpretation, the risk score was used to categorize the population into 2 subgroups based on the average value. All statistical analyses were performed in R 3.4.3 software.


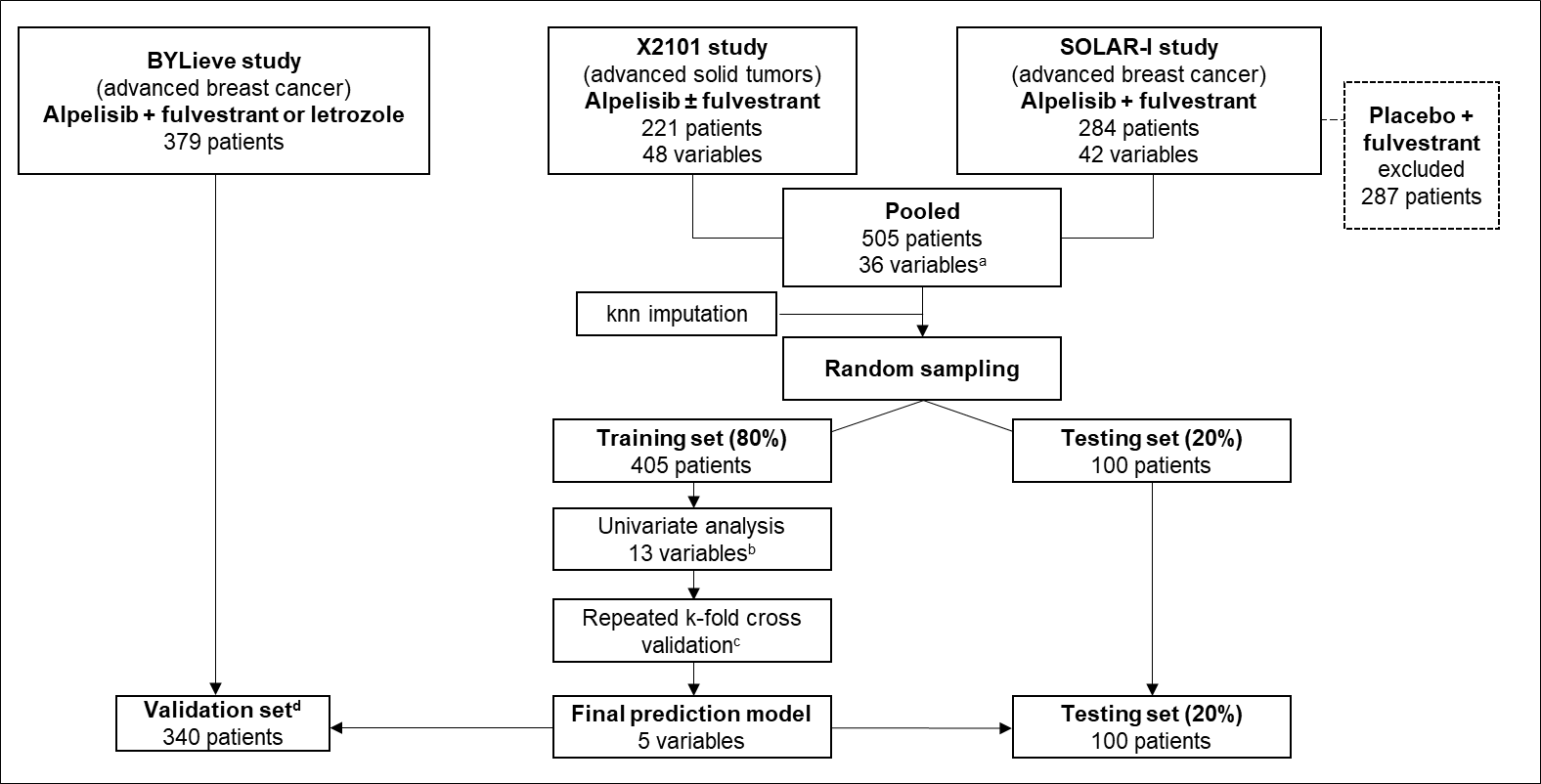
**Supplement Figure 1.** Flowchart of signature derivation from X2101 and SOLAR-I

^a^ Baseline variables present in the 2 studies with <20% missing. ^b^ Variables selected from univariate analysis while controlling false discovery rate at 10% using Benjamini and Hochberg procedure or known risk factors for diabetes. ^c^ Two-fold cross-validation repeated 20 times for elastic net, random forest, neural network, linear model with stepwise variable selection, and gradient-boosted decision trees. ^d^ Missing baseline data in the validation set are excluded.


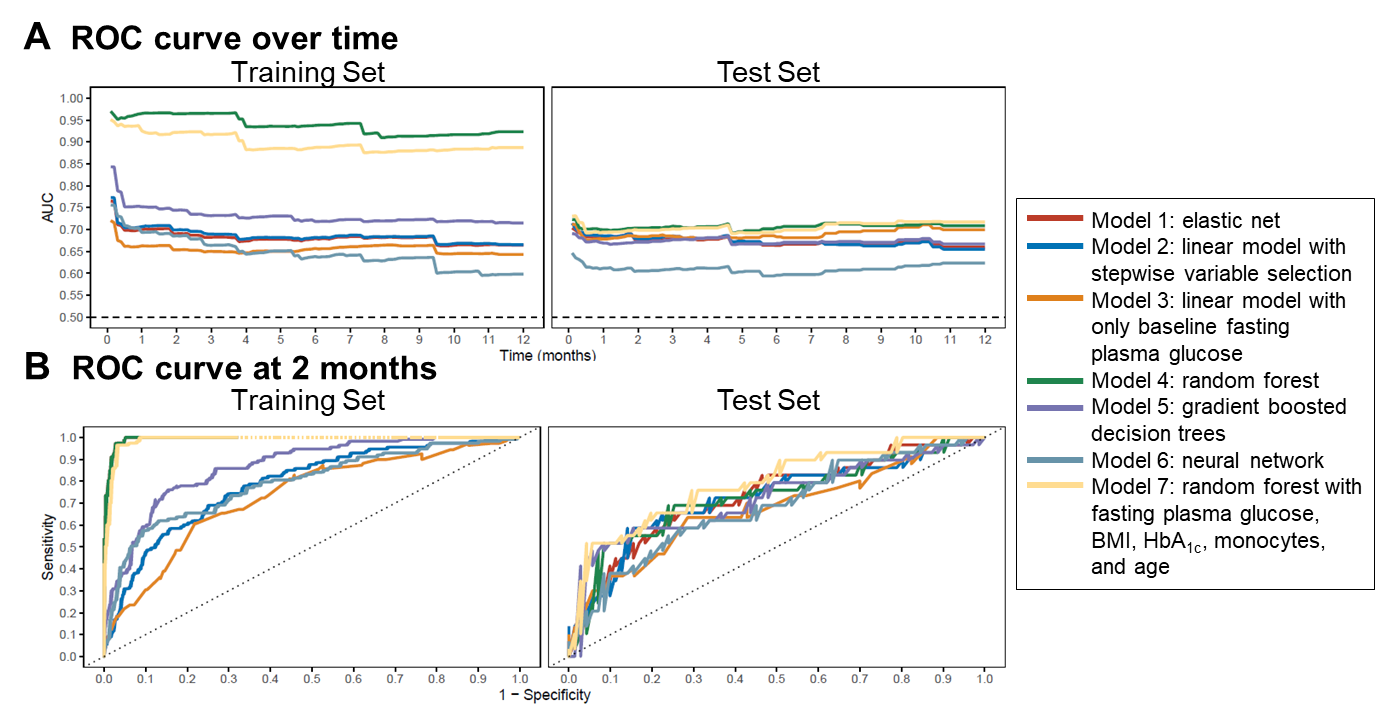
**Supplement Figure 2.** ROC curve over time (A) and at 2 months (B) of models to predict patients at risk of grade 3/4 hyperglycemia event

Time-dependent curves analysis of different models in the training set (left, 80% of the pool data) and test set (right, the 20% remaining sample).

AUC, area under the curve; ROC, receiver operating characteristic.

**Supplement Figure 3.** Predicted 2-month risk of grade 3/4 hyperglycemia (model 7) vs risk score (A) and distribution of risk scores in the training set (B)


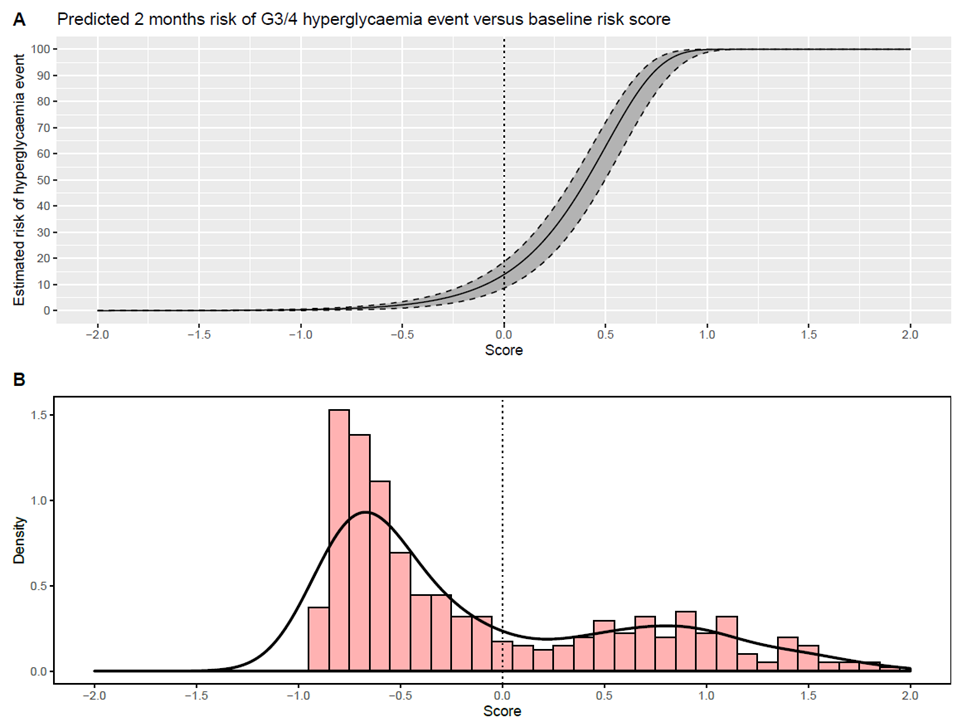


**Supplement Table 1.** Analysis of time to grade 3/4 hyperglycemia by risk group using Kaplan-Meier method using model 7

| Variable | **Training set** | | | **Test set** | | |
| --- | --- | --- | --- | --- | --- | --- |
|  | **Overall**  **(N=405)** | **Low risk (n=274)** | **High risk (n=131)** | **Overall (N=100)** | **Low risk (n=67)** | **High risk (n=33)** |
| No. of events | 129 (31.85%) | 3  (1.09%) | 126 (96.18%) | 32  (32%) | 12  (17.91%) | 20  (60.61%) |
| Median [95% CI], months | NE | NE | 0.49  [0.43-0.49] | NE | NE | 0.66  [0.49-NE] |
| Kaplan-Meier estimates rate [95% CI] at 3 months, % | 29.7  [24.9-34.1] | 0.4  [0-1.1] | 88.7  [81.7-93.1] | 29.6  [19.9-38.1] | 15.8  [6.2-24.4] | 57.6  [36.9-71.5] |
| Kaplan-Meier estimates rate [95% CI] at 2 months, % | 28.7  [24-33] | 0.4  [0-1.1] | 86.2  [78.7-91] | 29.6  [19.9-38.1] | 15.8  [6.2-24.4] | 57.6  [36.9-71.5] |
| Kaplan-Meier estimates rate [95% CI] at 1 month, % | 25.6  [21.2-29.8] | 0.4  [0-1.1] | 77.7  [69.3-83.8] | 25.1  [16.1-33.1] | 10.5  [2.8-17.6] | 54.5  [33.9-68.7] |

NE, value could not be estimated.

**Supplement Table 2.** Baseline characteristics of pooled studies, stratified by hyperglycemia risk

|  | **Overall (N=505)** | **Low risk (n=341)** | **High risk (n=164)** |
| --- | --- | --- | --- |
| **Age, years** |  |  |  |
| Minimum | 21 | 21 | 44 |
| Median (IQR) | 61 (54.00-67.00) | 60 (52.00-67.00) | 62.00 (56.75-70.00) |
| Mean ± SD | 60.34 ± 10.63 | 58.87 ± 11.11 | 63.40 ± 8.82 |
| Maximum | 87 | 87 | 82 |
| **BMI categories, kg/m^2^** |  |  |  |
| <18.5 | 17 (3.4%) | 12 (3.5%) | 5 (3.0%) |
| 18.5-25 | 207 (41.0%) | 170 (49.9%) | 37 (22.6%) |
| 25-30 | 156 (30.9%) | 100 (29.3%) | 56 (34.1%) |
| ≥30 | 112 (22.2%) | 51 (15.0%) | 61 (37.2%) |
| Missing | 13 (2.6%) | 8 (2.3%) | 5 (3.0%) |
| **Fasting plasma glucose categories, mmol/L** |  |  |  |
| <5.6 | 301 (59.6%) | 256 (75.1%) | 45 (27.4%) |
| 5.6-7 | 184 (36.4%) | 76 (22.3%) | 108 (65.9%) |
| ≥7 | 10 (2.0%) | 1 (0.3%) | 9 (5.5%) |
| Missing | 10 (2.0%) | 8 (2.3%) | 2 (1.2%) |
| **HbA_1c_ categories, %** |  |  |  |
| <5.7 | 296 (58.6%) | 229 (67.2%) | 67 (40.9%) |
| 5.7-6.5 | 172 (34.1%) | 90 (26.4%) | 82 (50.0%) |
| ≥6.5 | 15 (3.0%) | 4 (1.2%) | 11 (6.7%) |
| Missing | 22 (4.4%) | 18 (5.3%) | 4 (2.4%) |
| **Monocytes, ×10^9^/L** |  |  |  |
| Minimum | 0.01 | 0.01 | 0.11 |
| Median (IQR) | 0.40 (0.30-0.54) | 0.40 (0.28-0.53) | 0.43 (0.33-0.56) |
| Mean ± SD | 0.45 ± 0.21 | 0.43 ± 0.20 | 0.48 ± 0.21 |
| Maximum | 1.35 | 1.28 | 1.35 |
| Unknown | 1/505 (0.2%) | 1/341 (0.3%) | 0/164 (0.0%) |

IQR, interquartile range.
